# Mulberry Leaf Polysaccharides Attenuate Oxidative Stress Injury in Peripheral Blood Leukocytes by Regulating Endoplasmic Reticulum Stress

**DOI:** 10.3390/antiox13020136

**Published:** 2024-01-23

**Authors:** Wenqiang Jiang, Yan Lin, Linjie Qian, Siyue Lu, Huaishun Shen, Xianping Ge, Linghong Miao

**Affiliations:** 1 Wuxi Fisheries College, Nanjing Agricultural University, Wuxi 214081, China; 2020213006@stu.njau.edu.cn (W.J.); 2022213003@stu.njau.edu.cn (L.Q.); gexp@ffrc.cn (X.G.); 2 Key Laboratory of Freshwater Fisheries and Germplasm Resources Utilization, Ministry of Agriculture and Rural Affairs, Freshwater Fisheries Research Center, Chinese Academy of Fishery Sciences, Wuxi 214081, China; liny@ffrc.cn (Y.L.); lusiyue@ffrc.cn (S.L.)

**Keywords:** mulberry leaf polysaccharides, peripheral blood leukocytes, oxidative damage, endoplasmic reticulum stress, *Megalobrama amblycephala*

## Abstract

The present study assessed the protective effects and underlying mechanisms of mulberry leaf polysaccharides (MLPs) against hydrogen peroxide (H_2_O_2_)-induced oxidative stress injury in the peripheral blood leukocytes (PBLs) of *Megalobrama amblycephala*. Five treatment groups were established in vitro: the NC group (PBLs incubated in an RPMI-1640 complete medium for 4 h), the HP group (PBLs incubated in an RPMI-1640 complete medium for 3 h, and then stimulated with 100 μM of H_2_O_2_ for 1 h), and the 50/100/200-MLP pre-treatment groups (PBLs were pre-treated with MLPs (50, 100, and 200 μg/mL) for 3 h, and then stimulated with 100 μM of H_2_O_2_ for 1 h). The results showed that MLP pre-treatment dose-dependently enhanced PBLs’ antioxidant capacities. The 200 μg/mL MLP pre-treatment effectively protected the antioxidant system of PBLs from H_2_O_2_-induced oxidative damage by reducing the malondialdehyde content and lactic dehydrogenase cytotoxicity, and increasing catalase and superoxide dismutase activities (*p* < 0.05). The over-production of reactive oxygen species, depletion of nicotinamide adenine dinucleotide phosphate, and collapse of the mitochondrial membrane potential were significantly inhibited in the 200-MLP pre-treatment group (*p* < 0.05). The expressions of endoplasmic reticulum stress-related genes (forkhead box O1α (*foxO1α*), binding immunoglobulin protein (*bip*), activating transcription factor 6 (*atf6*), and C/EBP-homologous protein (*chop*)), Ca^2+^ transport-related genes (voltage-dependent anion-selective channel 1 (*vdac1*), mitofusin 2 (*mfn2*), and mitochondrial Ca^2+^ uniporter (*mcu*)), and interleukin 6 (*il-6*) and bcl2-associated x (*bax*) were significantly lower in the 200-MLP pre-treatment group than in the HP group (*p* < 0.05), which rebounded to normal levels in the NC group (*p* > 0.05). These results indicated that MLP pre-treatment attenuated H_2_O_2_-induced PBL oxidative damage in the *M. amblycephala* by inhibiting endoplasmic reticulum stress and maintaining mitochondrial function. These findings also support the possibility that MLPs can be exploited as a natural dietary supplement for *M. amblycephala,* as they protect against oxidative damage.

## 1. Introduction

Concurrent with the rapid development of the intensive farming mode, the aquaculture environment has been worsening. Exposure of fish to various unfavorable environmental factors leading to fish disease epidemics [1]. The use of chemical synthetic drugs has increased with the development of science and technology. Eventually, the toxic effects of these chemical synthetic drugs became evident against animals, including aquatic animals. This triggered scientists to start searching for natural products with immune-enhancing properties that could be used as dietary supplements [2]. The physiological effects of polysaccharides as a natural dietary supplement are mainly reflected through their immunomodulatory, immune-enhancing adjuvant, and antiviral effects. Large yellow croaker (*Larimichthys crocea*) macrophages pre-treated with *Astragalus* polysaccharides counteracted inactivated *Vibrio alginolyticus*-induced inflammatory injury by attenuating reactive oxygen species (ROS) production and inhibiting the release of inflammatory factors [3]. The common carp (*Cyprinus carpio*) fed with dietary *Dandelion* polysaccharides exhibited enhanced growth performance, suppressed serum oxidative stress, and increased resistance to an *Aeromonas hydrophila* challenge [2]. *Codonopsis pilosula* polysaccharides are a crucial immune enhancer that significantly increased non-specific immunity and resistance against white spot syndrome virus infection in crayfish (*Procambarus clarkia*) [4].

Mulberry (*Morus alba* L.) is widely distributed between 50° N and 10° S at altitudes below 4000 m, and parts of this plant have medicinal and edible properties [5]. Polysaccharides isolated from the leaves, fruits, branches, roots, and bark of mulberry exhibit antidiabetic activity, immunomodulatory activity, antioxidant activity, hepatoprotective and renoprotective activities, etc. [6,7,8,9]. Few studies have investigated the effects of mulberry leaf polysaccharides (MLPs) than the effects of polysaccharides from the remaining parts of mulberry, especially in aquatic animals [5]. The diphenyl-picrylhydrazyl (DPPH) radical scavenging rate of 1.5 mg/mL of *Thymus vulgaris* leaf polysaccharide (DPPH, 92.0 ± 0.5%) was comparable to that of standard antioxidants, butylated hydroxyanisole (95.7 ± 0.3%) and butylated hydroxytoluene (96.6 ± 0.4%) [10]. The scavenging rates of hydroxyl radicals and DPPH were 94.8% and 93.4% for *Althaea officinalis* leaf polysaccharides at 20 mg/mL with antimicrobial activity [11]. Like other leaf polysaccharides, MLPs have powerful DPPH, hydroxyl radical scavenging rates; MLPs mediate the immune response by regulating cytokine and immunoglobulin levels, thus exerting immunomodulatory effects [12]. Body weight and liver damage were recovered in cyclophosphamide-injured mice after being fed an MLP-supplemented diet [13]. MLPs mitigated oxidative stress in type 2 diabetic mice by repairing their mitochondrial function and inhibiting the release of inflammatory factors [14,15]. MLPs enhanced the antioxidant capacity (SOD, CAT, and GSH) in diabetic rats in response to diabetes-induced oxidative stress [16]. However, fewer studies have been conducted on the mechanism of action by which MLPs exert their functions and their effects on signaling between organelles. Moreover, the immunomodulatory and antioxidant benefits of MLPs are neither a single receptor nor a single signaling pathway, but have a variety of targets and pathways. Therefore, studying the main targets or pathways of MLPs in vitro is particularly important.

Oxidative stress implies an imbalance between ROS and antioxidant systems. Excessive ROS production causes oxidative stress and chronic inflammation, thereby leading to impaired cellular function. The endoplasmic reticulum (ER) is a vital membrane organelle in eukaryotic cells, including two types of smooth ER and rough ER [17]. The smooth ER is the intracellular Ca^2+^ reservoir that controls the regulation of intracellular Ca^2+^ homeostasis. The rough ER is involved in functions such as protein synthesis, transport, modification, and folding [18,19]. Maintaining ER homeostasis is vital for regular cellular physiological activity, as the particular environment of the ER is highly sensitive to oxidative stress, and unfolded/misfolded/mutated proteins may accumulate in the ER [20,21]. Tissue damage occurs if the unfolded protein reaction (UPR) exceeds self-compensating [22]. ER stress (ERS) and UPR activation are crucial in inducing aquatic diseases [23,24]. Studies have shown that *Achyranthes bidentata* polysaccharides, *Astragalus* polysaccharides, *Lycium barbarum* polysaccharides, etc., alleviate ERS at the protein or gene level [25,26,27]. However, the mechanism underlying the effect of MLPs on ERS is yet to be clarified.

Peripheral blood leukocytes (PBLs) are a critical class of blood cells known as immune cells [28]. Fish PBLs can phagocytose xenobiotics, produce antibodies, resist pathogen invasion, etc. [29]. When activated by stress, PBLs migrate inside and outside the blood vessels, mediating the inflammatory process and enhancing immunity [30]. Therefore, in this study, PBLs of *M. amblycephala* were pre-treated with MLPs and thereafter stimulated with a hydrogen peroxide (H_2_O_2_) solution in vitro to investigate the protective effect of MLPs on oxidative stress damage.

## 2. Materials and Methods

### 2.1. MLPs

The MLPs were purchased from Yangling Ciyuan Company (Yangling, China).

#### 2.1.1. Determination of Molecular Weights

The molecular weight of the MLPs was evaluated through gel permeation chromatography (GPC, ELEOS, Wyatt). The molecular weight distribution was calculated using the GPC software. Chromatographic separation was executed using a Shodex OHpak^®^ SB-806 column (Showa Denko, Minato-ku, Tokyo, Japan) (300 mm × 8.0 mm, 13 μm) connected in series with a Shodex OHpak^®^ SB-803 column (Showa Denko, Minato-ku, Tokyo, Japan) (300 mm × 8.0 mm, 6 μm). The mobile phase employed was H_2_O + 0.02% NaN_3_ at a 1.0 mL/min flow rate. The column oven temperature was kept at 40 °C. A 500 μL injection volume was utilized.

#### 2.1.2. Monosaccharide Composition Analysis

The monosaccharide composition of MLPs was determined through high-performance liquid chromatography. (1) Pre-treatment: An appropriate amount of the sample was taken in a hydrolysis tube; 4 mol/L of TFA (trifluoroacetic acid) was added to 1 mL, and hydrolyzed at 120 °C for 2 h. After removing the sample, it was blow-dried under nitrogen. (2) Derivatization reaction: 1 mL of a 0.5 mol/L PMP–methanol solution, and 0.5 mL of a 0.3 mol/L NaOH solution was added to the blow-dried sample in a 70 °C water bath for 60 min, then cooled. Furthermore, 0.5 mL of a 0.3 mol/L HCl solution was added, followed by 0.5 mL of chloroform, shaken well, and left to stand for 20 min; the lower layer was discarded. The aqueous layer was needed after being extracted three times. (3) An analysis of the samples obtained in step (2) was carried out using a Shiseido-C18 column (Shiseido, Chuo Ward, Tokyo, Japan) (4.6 × 250 mm. 5 μm) at 25 °C. The mobile phase consisted of 0.1 M KH_2_PO_4_ (pH 6.8) and acetonitrile (82:18 (*v*/*v*)) at a 1.0 mL/min flow rate. The samples were detected at a wavelength of 245 nm, and the injection volume was set at 10 μL.

### 2.2. PBL Isolation

PBLs were collected from healthy *M. amblycephala* ‘Huahai No.1′ (200 g ± 20 g). Before the blood was sampled, the fish were quickly anesthetized with 100 mg/L of MS-222 (Sigma, Saint Louis, MO, USA). Then, a 1% potassium permanganate solution was used to disinfect the fish through immersion for 10 min, and 2 mL of blood was collected from the tail vein of each fish under a sterile environment by using syringes containing a sodium heparin anticoagulant (500 U/mL, Solarbio Technology Co., Ltd., Beijing, China). The blood samples were mixed and diluted with a triple volume of an RPMI-1640 complete medium (RPMI-1640 medium containing 1% penicillin–streptomycin solution (100×, Solarbio Technology Co., Ltd., Beijing, China) and 10% fetal bovine serum (Sijiqing Biological Engineering Materials Co., Ltd., Hangzhou, China)). Then, 5 mL of diluted blood was spread flat on the surface of an equal amount of Ficoll Plus 1.077 isolate (Solarbio Technology Co., Ltd., Beijing, China). Following centrifugation at 850× *g* for 25 min, leukocyte strips placed between the plasma and the isolate were collected using a Pasteur pipette and washed twice with the RPMI-1640 complete medium. The Typan blue method (Beyotime Biotechnology Inc., Shanghai, China) was used to detect cell viability to make sure that the leukocyte viability was >95% [31]. Live PBLs were counted using a hemocytometer plate and adjusted to 6 × 10^6^ cells/mL of the cell suspension. The PBLs were incubated under a 5% CO_2_ atmosphere at 27 °C and 95% humidity.

### 2.3. Experimental Treatment and Sampling

Different concentrations of the MLP working solution (100, 200, and 400 µg/mL) were prepared using the RPMI-1640 complete medium. Then, 6 × 10^6^ cells/mL of the cell suspension was mixed with equal amounts of the MLP working solution. The final concentration of the cell suspension was 3 × 10^6^ cells/mL, with MLP concentrations of 50, 100, and 200 µg/mL. The H_2_O_2_ working solution was obtained through gradient dilution of a 9.8 M H_2_O_2_ solution (detected at 240 nm, Sinopharm Chemical Reagent Co., Ltd., Shanghai, China) to 1.1 mM by using the RPMI-1640 complete medium. The H_2_O_2_ concentration of the cell suspension was adjusted to 100 µM by adding 10 µL of the H_2_O_2_ working solution per 100 µL of the cell suspension.

The experimental groups were classified as per the five treatments administered. For the negative control (NC) group, the PBLs were incubated alone in the RPMI-1640 complete medium for 4 h. In the H_2_O_2_ treatment (HP) group, the PBLs were incubated in the RPMI-1640 complete medium alone for 3 h and then co-treated with 100 µM of H_2_O_2_ for 1 h. In the MLP pre-treatment groups (remarked as 50-MLP, 100-MLP, and 200-MLP, respectively), PBLs were pre-treated with different MLP concentrations (50, 100, and 200 µg/mL) for 3 h, respectively, and then co-treated with 100 µM of H_2_O_2_ for 1 h.

### 2.4. Chemical Analyses

#### 2.4.1. Determination of PBLs’ Antioxidant Capacity

First, 3 mL of the 3 × 10^6^ cells/mL PBL suspension was seeded into 6-well plates. Three wells were combined into one replicate, and a total of six replicates were collected to detect superoxide dismutase (SOD) and catalase (CAT) activities; total protein, malondialdehyde (MDA), and nicotinamide adenine dinucleotide phosphate (NADPH) contents; and lactic dehydrogenase (LDH) cytotoxicity by using commercial kits in strict compliance with the instructions (Beyotime Biotechnology Inc., Shanghai, China).

The ROS and mitochondrial membrane potential (MMP) levels of the PBLs were determined using the 2,7-dichlorodihydrofluorescein diacetate (DCFH-DA) method [32] and the 5,5′,6,6′-Tetrachloro-1,1′,3,3′-tetraethyl-imidacarbocyanine iodide (JC-1) method [33], respectively, following the instructions provided in the commercial kits (Beyotime Biotechnology Inc., Shanghai, China). The cellular ROS and MMP fluorescence intensities were detected under a fluorescence microscope (Leica DM2500 LED, Wetzlar, Germany). These results were expressed as the fluorescence intensity, which was analyzed using Image J 1.8.0 (National Institutes of Health, Bethesda, ML, USA).

#### 2.4.2. Real-Time PCR Analysis (qRT-PCR)

Six wells were combined into one replicate, and thus, nine replicates were collected for gene expression assays. Total RNA was extracted from the PBLs of *M. amblycephala* by using the RNAiso Plus kit (TaKaRa Biomedical Technology Co., Ltd., Dalian, China). For cDNA synthesis, a 400 ng/µL RNA sample was reverse transcribed into cDNA using the PrimeScript™ RT reagent Kit with a gDNA Eraser (TaKaRa Biomedical Technology Co., Ltd., Dalian, China). Genes expression was determined using qRT-PCR (CFX96, Bio-Rad, Hercules, CA, USA) with the TB Green™ Premix Ex Taq™ II (TaKaRa Biomedical Technology Co., Ltd., Dalian, China). Table 1 presents the sequences of gene-specific primers used. The qRT-PCR procedure details are as follows: 95 °C, 30 s; 95 °C, 5 s, 60 °C, 30 s (39 cycles); and extension at 95 °C, 10 s. The 2^−ΔΔCT^ method was used to calculate gene expressions. The expression for each gene was normalized to that of beta-cytoskeletal actin (*β-actin*).

### 2.5. Statistical Analyses

Data were analyzed using the SPSS 22.0 software (SPSS Inc., Chicago, IL, USA) after determining that the two assumptions of normality and uniformity of variance were met. A one-way analysis of variance, Tukey’s test, and an independent *t*-test were performed to compare the differences between various groups. Data are presented as the mean ± SEM. Differences were considered statistically significant at *p* < 0.05.

## 3. Results

### 3.1. Monosaccharide Composition and Molecular Weights of MLPs

**Figure 1:**
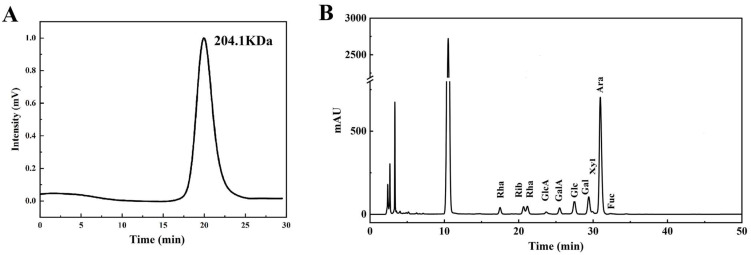
Physicochemical properties of mulberry leaf polysaccharides. Molecular weight distribution of mulberry leaf polysaccharides (**A**). Monosaccharide composition of mulberry leaf polysaccharides (**B**).

Figure 1 presents the physicochemical properties of the MLPs. The molecular weight of the MLPs was 204.1 kDa (Figure 1A). The monosaccharide composition detected in MLPs included mannose, ribose, rhamnose, glucuronic acid, galacturonic acid, glucose, galactose, xylose, arabinose, and fucose at a molar ratio of 2.65:4.30:5.84:1.48:4.11:5.28:10.64:1.36:63.78:0.56 (Figure 1B).

### 3.2. Effect of MLPs on the Antioxidant Capacity of PBLs Exposed to H_2_O_2_

**Figure 2:**
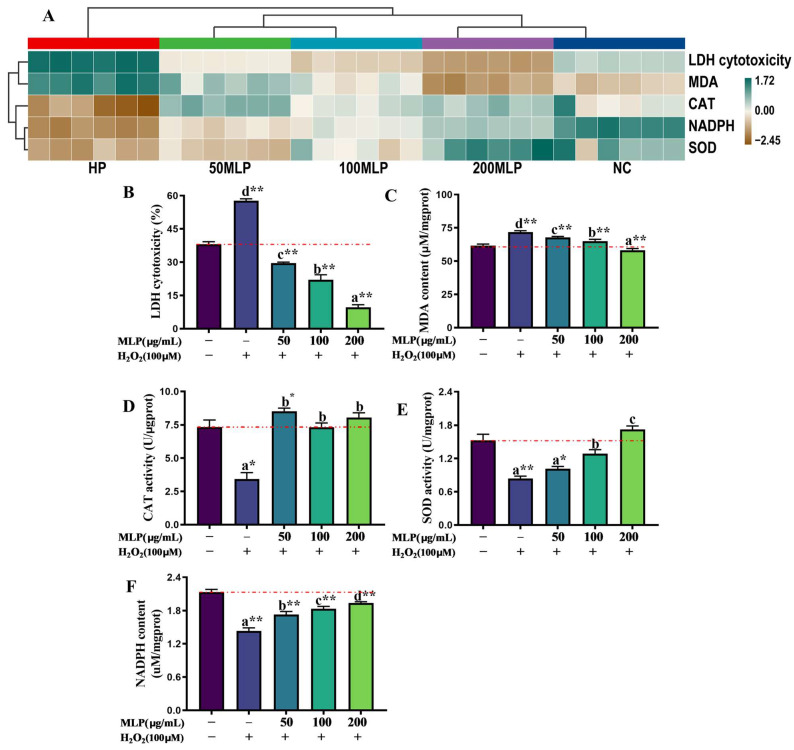
The effect of MLPs on the antioxidant capacity of H_2_O_2_-exposed PBLs. (**A**) Visualizing LDH cytotoxicity, MDA and NADPH contents, and CAT and SOD activities on a pair heatmap. In the graphical presentation of data, numerical values are displayed by colors. The dendrogram for strain clustering is shown on the top and left sides of the heatmap. The width of the cluster merged from the two sides represents the distance of the two clusters. LDH cytotoxicity (**B**), MDA content (**C**), CAT activity (**D**), SOD activity (**E**), NADPH content (**F**). Data are expressed as the mean ± SEM (*n* = 6). Different little letters above the bars indicate significant differences (*p* < 0.05, Tukey’s test) among the HP group and MLP (50-MLP, 100-MLP, and 200-MLP) groups. * indicates a significant difference between the NC and HP, MLP (50-MLP, 100-MLP, and 200-MLP) groups (*p* < 0.05, independent *t*-test); ** indicates an extremely significant difference between the NC and HP, MLP (50-MLP, 100-MLP, and 200-MLP) groups (*p* < 0.01, independent *t*-test). ‘−’ indicates no reagents added; ‘+’ indicates reagents added.

Treatment visual clustering was performed to determine the differences in antioxidant indicators (NADPH and MDA contents, SOD and CAT activities, and LDH cytotoxicity) by using a single-heatmap method (Figure 2A). The 200 μg/mL MLP pre-treatment group, which exhibited enhanced antioxidant capacities of PBLs exposed to H_2_O_2_, were clustered into one category with the NC group.

Specifically, compared with the NC group, the MDA content and LDH cytotoxicity in PBLs were significantly higher because of H_2_O_2_ exposure (independent *t*-test, *p* < 0.01, Figure 2B,C). LDH cytotoxicity in PBLs was inhibited significantly in the MLP pre-treatment (50, 100, and 200 μg/mL) groups compared with the NC group (independent *t*-test, *p* < 0.01), and LDH cytotoxicity decreased remarkably in a dose-dependent manner with increasing MLP concentrations from 0 μg/mL to 200 μg/mL after H_2_O_2_ exposure (Tukey’s test, *p* < 0.05). Moreover, the MDA contents of the MLP pre-treatment groups exhibited a significant dose-dependent reduction compared with that of the HP group (Tukey’s test, *p* < 0.05). The MDA content was significantly lower in the 200-MLP pre-treatment group than in the NC group (independent *t*-test, *p* < 0.01).

Compared with the NC group, the SOD and CAT activities and NADPH content in the HP group significantly decreased (independent *t*-test, *p* < 0.01; Figure 2D–F). However, the SOD and CAT activities and NADPH content were enhanced in the MLP pre-treatment groups to a different extent relative to the HP group (Tukey’s test, *p* < 0.05). MLP pre-treatment (50, 100, and 200 μg/mL) remarkably improved the CAT activities before H_2_O_2_ stress (Tukey’s test, *p* < 0.05). Furthermore, CAT activity significantly increased in the 50-MLP pre-treatment group compared with the NC group (independent *t*-test, *p* < 0.05). In addition, compared with the HP group, the SOD activity and NADPH content were dose-dependently elevated to the levels in the MLP pre-treatment (50, 100, and 200 μg/mL) groups responding to H_2_O_2_ exposure. The SOD activities in the 100-MLP and 200-MLP pre-treatment groups were higher than those in the HP group and 50-MLP pre-treatment group (Tukey’s test, *p* < 0.05). Although the NADPH contents in the MLP pre-treatment (50-MLP, 100-MLP, and 200-MLP) groups were not recovered to those in the NC group (independent *t*-test, *p* < 0.01), noticeable increases were observed compared with the HP group (Tukey’s test, *p* < 0.05).

### 3.3. Assessment of ROS Generation and the MMP Status

**Figure 3:**
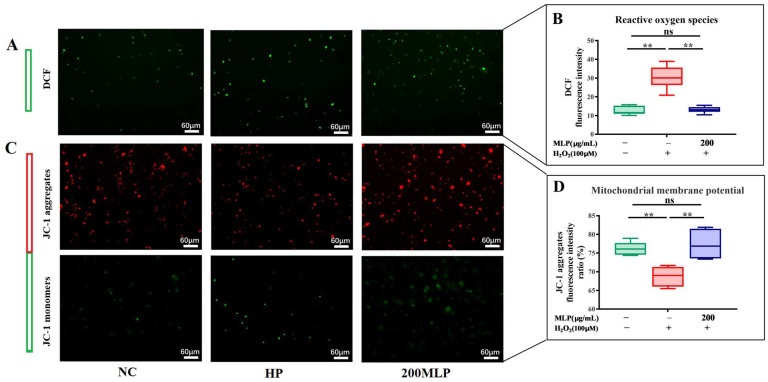
Effect of MLPs on ROS generation (**A**,**B**) and MMP status (**C**,**D**) in H_2_O_2_-induced PBLs. Data are expressed as the mean ± SEM (*n* = 6); ns indicates no significant difference (independent *t*-test, *p* > 0.05); * indicates a significant difference (independent *t*-test, *p* < 0.05); ** indicates an extremely significant difference (independent *t*-test, *p* < 0.01). ‘−’ indicates no reagents added; ‘+’ indicates reagents added.

The ROS and MMP levels were significantly enhanced and reduced in the HP group compared with the NC group (independent *t*-test, *p* < 0.01, Figure 3). However, the 200 μg/mL MLP pre-treatment reversed the ROS and MMP levels responding to H_2_O_2_ exposure (independent *t*-test, *p* < 0.01). No significant difference in ROS and MMP levels was observed between the NC group and 200-MLP pre-treatment group (independent *t*-test, *p* > 0.05).

### 3.4. Assessment of Gene Expression

**Figure 4:**
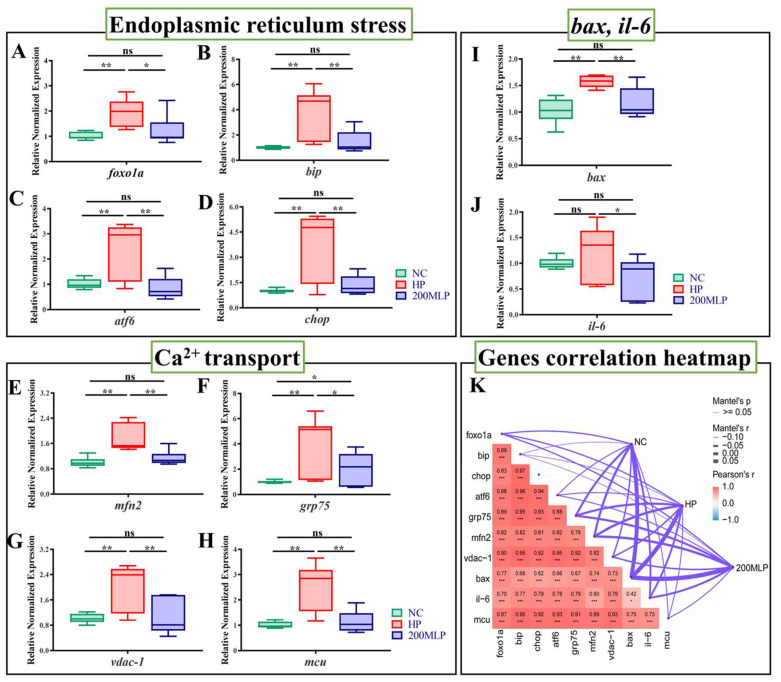
Assessment of gene expressions: *foxO1α* (**A**), *bip* (**B**), *atf6* (**C**), *chop* (**D**), *mfn2* (**E**), *grp75* (**F**), *vdac-1* (**G**), *mcu* (**H**), *bax* (**I**), and *il-6* (**J**). Data are expressed as the mean ± SEM (*n* = 9); ns indicates no significant difference (independent *t*-test, *p* > 0.05); * indicates a significant difference (independent *t*-test, *p* < 0.05); ** indicates an extremely significant difference (independent *t*-test, *p* < 0.01). Heatmap illustrating the relationship between genes (**K**). Rows and columns correspond to genes; each cell contains the corresponding correlation and *p* value. Pearson’s R-values are color-coded according to the color legend. The edge width corresponds to Mantel’s r statistic for the corresponding distance correlations between the different diets and genes. * indicates *p*-value < 0.05, ** indicates *p*-value < 0.01, *** indicates *p*-value < 0.001 (**K**).

As shown in Figure 4, compared with the NC group, the expressions of *foxO1α*, *bip*, *atf6*, *chop*, *mfn2*, *grp75*, *vdac-1*, *mcu*, and *bax* were remarkably up-regulated after H_2_O_2_ exposure (independent *t*-test, *p* < 0.01, Figure 4A–I), but significantly down-regulated when pre-treated with 200 μg/mL of MLPs before H_2_O_2_ exposure (independent *t*-test, *p* < 0.05). PBLs pre-treated with 200 μg/mL of MLPs before H_2_O_2_ exposure remarkably inhibited the up-regulation of *il*-*6* expression (independent *t*-test, *p* < 0.05, Figure 4J). In addition, no difference was observed between the NC group and 200-MLP pre-treatment group in the expressions of *foxO1α*, *bip*, *atf6*, *chop*, *mfn2*, *vdac-1*, *mcu*, *bax*, and *il-6* (independent *t*-test, *p* > 0.05). *Grp75* expression in the 200-MLP pre-treatment group was significantly enhanced relative to the NC group (independent *t*-test, *p* < 0.05). The correlation coefficient matrix visualization analysis presented a significant positive correlation between the expressions of *foxO1α*, *bip*, *atf6*, *chop*, *vdac1*, *grp75*, *mfn2*, *mcu*, *il-6*, and *bax* (Figure 4K). The network plot revealed that the NC, HP, and 200-MLP pre-treatment groups correlated more strongly with *bax* expression.

## 4. Discussion

H_2_O_2_ has been used in cell biology studies to induce ERS for building cellular damage models [34,35]. In aquatic animal studies, *bip* and *chop* expressions, two important ERS markers, were up-regulated in the gills, muscles, and heart of H_2_O_2_-stimulated common carp (*C. carpio*) [36]. In the present study, *bip*, *atf6*, and *chop* expressions were significantly increased in the HP group than in the NC group, which is consistent with the results of previous studies [35,36]. Under ERS, the ER chaperone molecule *bip* dissociates from the ER transmembrane receptor *atf6*, activating the downstream UPR signal [37]. The microphage nuclear factor kappa B (*nf-κb*) signaling pathway is activated by *atf6*, which elevates the contents of pro-inflammatory factors such as IL-6 and tumor necrosis factor-α [38]. The *Atf6/chop* pathway has a vital regulatory role in ERS [39]. Bioactive compounds can reduce ERS levels and improve stress resistance. Emodin reduced hepatic ERS (*bip*, *chop*, and *atf6*) levels in Gibel carp (*Carassius gibelio*) and protected the liver from acute hypoxic stress-induced oxidative damage [40]. Meanwhile, at the protein level, *L. barbarum* polysaccharides reduced tunicamycin-induced ERS in IPEC-J2 cells, thereby protecting the cells against ERS-induced apoptosis [41]. In our study, MLP pre-treatment increased cell resistance to H_2_O_2_-induced ERS and down-regulated *foxO1α*, *bip*, *atf6*, *chop*, and *il-6* expressions. The proteins forkhead box O1 (*foxO1*) and forkhead box O3 (*foxO3*) are activated during high levels of oxidative stress [42,43], which is similar to the significant augment in PBLs’ *foxO1α* expression observed in the HP group. Forkhead transcription factors recognize ERS, and *foxO1* suppression might restrain ERS [44,45]. Polysaccharide-enhanced stress resistance in aquatic animals has been reported in orange-spotted grouper (*Epinephelus coioides*), hybrid snakeheads (*Channa maculata ♀ × Channa argus ♂*), Nile tilapia *(Oreochromis niloticus)*, etc. [46,47,48,49]. The immunological properties of MLPs are related to their origin, structure, composition, water solubility, and molecular weight [50]. The molecular weight of MLPs in our study was 204.1 kDa. Polysaccharides usually require their molecular weight to be in the 10–350 kDa range to exert immunomodulatory activity [51]. *Radix Puerariae lobatae* polysaccharides (molecular weight: 10.43 kDa) had a strong free radical scavenging ability in vitro, and enhanced the hepatic antioxidant capacity and inhibited the release of pro-inflammatory factors in alcoholic liver disease mice [52]. *Callicarpa nudiflora* Hook polysaccharides (molecular weight: 31.38 kDa) inhibited intestinal inflammation in ulcerative colitis mice by affecting the *nf-κb*/*mapk* pathway [53]. Regarding the monosaccharide composition [5], the molar mass of arabinose and galactose following MLP hydrolysis in our experiment was higher than that of the other monosaccharides. Scholars have found that arabinose and galactose in polysaccharides have stronger immune activity than the other monosaccharides [54,55]. The anti-inflammatory activity of *L. barbarum* polysaccharides [55] and *Helicteres angustifolia* L polysaccharides [56] was significantly enhanced by high levels of arabinose and galactose, which enhanced the nitric oxide level and phagocytosis of macrophages RAW264.7.

ERS is an unfolded response of proteins that also manifests as Ca^2+^ disorders within the ER. The ER is a major organelle for Ca^2+^ storage, while mitochondria are the main players in intracellular Ca^2+^ uptake and apoptosis regulation, with Ca^2+^ flow regulated between these two organelles by mitochondria-associated membranes (MAMs) [57]. Consequently, when Ca^2+^ levels in the ER are disturbed, Ca^2+^ may be released directly from the ER into the cytoplasm and possibly into the mitochondria through MAMs [58]. MAMs represent a ‘bridge’ between mitochondria and ER, while *mfn2* and *grp75* are crucial genes regulating this physical linkage, playing a major role in Ca^2+^ transport [59]. Over-expressions of *mfn2* and *grp75* in duck renal tubular epithelial cells led to excessive entry of Ca^2+^ into mitochondria, thereby stimulating the release of pro-apoptotic factors and inducing apoptosis [58]. Consistent with the HP group, *mfn2*, *grp75*, *vdac-1*, *mcu*, and *bax* expressions were significantly increased compared with the NC group. *Grp75* links Ca^2+^ channels (located in the ER membrane) and *vdac-1* (located in the outer mitochondrial membrane) to transport Ca^2+^ into the mitochondria, and through *mcu* to the mitochondrial matrix [60]. Mitochondrial Ca^2+^ overload-induced apoptosis is caused by *mfn2* over-expression by promoting a massive influx of Ca^2+^ from the ER into the mitochondria [61]. The present study revealed that mitochondrial Ca^2+^ overload in the HP group induces ROS over-production and MMP decrease, possibly opening the mitochondrial permeability transition pore or even evolving into an irreversible and excessive opening, releasing apoptotic proteins [62,63]. Polysaccharides exert immunomodulatory [64], anti-aging [65], anti-apoptosis [66], and anti-hypertrophic [67] effects by regulating cell Ca^2+^ contents. A *Sparassis crispa* polysaccharide pre-treatment resisted L-glutamate-induced damage by significantly reducing Ca^2+^ content and ROS accumulation in differentiated PC12 cells and attenuating MMP dissipation [68]. A *Cuscuta chinensis Lam* polysaccharide treatment reduced Ca^2+^ content, *bax* expression, and the apoptosis rate in SD rats compared with the D-galactose injury group, and inhibited apoptosis through the mitochondrial pathway [66]. Consistent with the aforementioned results, MLP pre-treatment exerted an anti-apoptosis effect by significantly reversing mitochondrial Ca^2+^ overload-induced mitochondrial dysfunction (ROS overproduction and MMP collapse) and decreased *bax* expression and LDH cytotoxicity. *Chop*-induced *bax* over-expression leads to the formed polymers being inserted into the outer mitochondrial membrane, thereby enhancing mitochondrial membrane permeability [69]. LDH is present in the cytoplasm and is commonly considered a cytotoxicity index, as it is released outside the cell during cell damage or death [70]. Our experimental results demonstrated that MLP pre-treatment maintains mitochondrial function and inhibits cell apoptosis by regulating Ca^2+^ flow from the ER to the mitochondria, thereby balancing the mitochondrial Ca^2+^ content during H_2_O_2_ exposure.

Mitochondria are considered as the primary source of ROS in cells [71]. Ca^2+^ is released from the ER and used by mitochondria through MAMs, possibly promoting mitochondrial ROS production through different mechanisms [72]. The NADPH electrons are transferred to O_2_ by NADPH oxidase enzymes, producing superoxide negative ions (O_2_^−^, i.e., ROS) [73]. In the present experiment, H_2_O_2_ stimulation significantly increased and decreased ROS levels and NADPH content in PBLs, respectively. Excessive ROS in PBLs leads to mitochondrial and cell membrane lipid peroxidation, whereas elevated MDA content reflects the exacerbated lipid peroxidation level [74]. In the present study, the decreased NADPH and increased MDA indicated that H_2_O_2_ induced oxidative damage in PBLs. Leaf polysaccharides such as *Thymus vulgaris leaf* polysaccharides and *Althaea officinalis* leaf polysaccharides had an extremely strong free radical scavenging ability in vitro [10,11]. Previous studies have shown that higher contents of arabinose and galactose enhanced the scavenging ability of *Achyranthis bidentatae radix* polysaccharides against hydroxyl radicals, DPPH, and O_2_^−^ in vitro [75]. Plant polysaccharides supplied as dietary supplements increased the antioxidant capacity of snakehead fish (*Channa argus*) and grass carp (*Ctenopharyngodon idellus*) by increasing SOD and CAT activities in the enzymatic antioxidant system [76,77]. Consistent with the results of these studies, a pre-treatment of MLPs with a higher content of arabinose and galactose increased the SOD and CAT activities of PBLs, SOD converted H_2_O_2_-induced superoxide negative ions (O_2_^−^) into endogenous H_2_O_2_ and O_2_, and finally, the endogenous H_2_O_2_ was decomposed into H_2_O and O_2_ by CAT, alleviating incomplete oxygen reduction to PBL damage [76,77].

## 5. Conclusions

**Figure 5:**
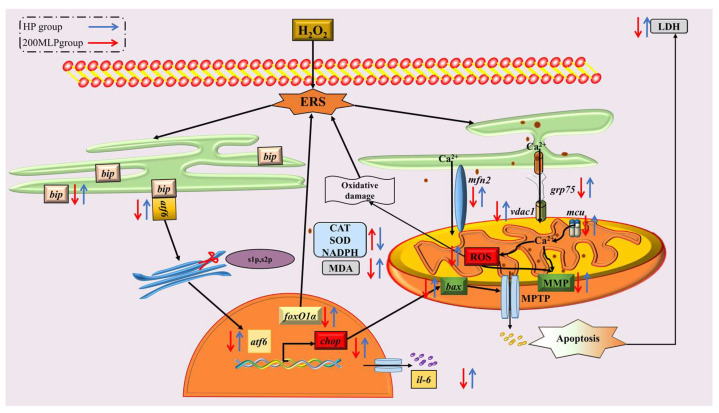
Schematic diagram depicting the effects and possible mechanisms of H_2_O_2_ stress on antioxidant capacity, endoplasmic reticulum stress, apoptosis, and Ca^2+^ transport-related gene expression. Notes: the blue arrow represents the HP group, and the red arrow represents the 200-MLP group; the upward arrows are representative of significant up-regulation or improvement, and the downward arrows indicate significant down-regulation or inhibition.

In conclusion, the present study indicated that MLP pre-treatment increased the antioxidant capacity of PBLs and enhanced their resistance to H_2_O_2_-induced oxidative stress. These protective effects were exerted through ERS resistance, inhibiting Ca^2+^ transport between the ER and mitochondria, maintaining the MMP potential, and reducing excess ROS production (Figure 5).

## Figures and Tables

**Table 1:** Primer sequences used for qRT-PCR.

Genes		Primer Sequence (5′-3′)	Product Length (bps)	Accession No.	Amplification Efficiency
*foxO1α*	Forward	GGATGATGCCTGAGATGG	148	XM_048206068.1	109.8%
	Reverse	GACCTGGAGGCTCGTGT			
*bip*	Forward	CCTGGGACAGAAGGTCACAC	133	XM_048186701.1	100.4%
	Reverse	AGCAGTTGGCTCGTTGATGA			
*atf6*	Forward	CGATCAGGATGGAGAGTGGGATA	157	XM_048207041.1	108.3%
	Reverse	AGGGCTACTCCACAATGGGT			
*chop*	Forward	ATGTGGTGCAGAGTTGGAGG	124	XM_048198700.1	108.2%
	Reverse	CACATCCAGAAACTCGGGCT			
*mcu*	Forward	AAGGGAGCGAAGAGAACACG	128	XM_048205815.1	106.6%
	Reverse	ATTTGCTGGACAGGGAGGTG			
*vdac1*	Forward	GATAACCTGGAGACGGCAGTT	146	XM_048166591.1	104.7%
	Reverse	GTATATCCCAGGCCGACGAG			
*mfn2*	Forward	TGCTCACTCTGGACTGCAAG	192	XM_048210720.1	109.0%
	Reverse	GACCGTCCTCTATGTGCCTG			
*grp75*	Forward	CGGCGTTATGATGACCCAGA	184	XM_048175758.1	106.7%
	Reverse	TCACAGCATGGCCCAAGTAG			
*il-6*	Forward	AAGACAACCGCACACTCGAT	122	XM_048203704.1	97.2%
	Reverse	CTGGGTCTCTTCACGCCTTT			
*bax*	Forward	AGTGTTTGCAGCAGATCGGA	162	XM_048196672.1	104.3%
	Reverse	AGAAAAGAGCCACCACCCTG			
*β-actin*	Forward	TCGTCCACCGCAAATGCTTCTA	190	AY170122.2	99.9%
	Reverse	CCGTCACCTTCACCGTTCCAGT			

Abbreviation: forkhead box O1α (*foxO1α*), binding immunoglobulin protein (*bip*), activating transcription factor 6 (*atf6*), C/EBP-homologous protein (*chop*), voltage-dependent anion-selective channel 1 (*vdac1*), glucose-regulated proteins 75 (*grp75*), mitofusin 2 (*mfn2*), mitochondrial Ca^2+^ uniporter (*mcu*), bcl2-associated x (*bax*), interleukin 6 (*il-6*).

## Data Availability

The authors confirm that the data supporting the findings of this study are available within the manuscript and table.

## References

[1] Huang K., Jiang L., Huang W., Li X., Yuan L., Jiang J., Zhou S., Wang Y., Xie J. (2023). Investigating the interplay between Cryptocaryon irritans ectoparasite infection and the immune responses of the head kidney in silver pomfret (*Pampus argenteus*). Aquaculture.

[2] Giri S.S., Kim S.G., Woo K.J., Jung W.J., Lee S.B., Lee Y.M., Jo S.J., Kim J.H., Park S.C. (2022). Impact of dandelion polysaccharides on growth and immunity response in common carp *Cyprinus carpio*. Fish Shellfish Immunol..

[3] Wang Y., Wu Z., Chen H., Liu R., Zhang W., Chen X. (2022). Astragalus polysaccharides protect against inactivated Vibrio alginolyticus-induced inflammatory injury in macrophages of large yellow croaker. Fish Shellfish Immunol..

[4] Liu F., Geng C., Qu Y.K., Cheng B.X., Zhang Y., Wang A.M., Zhang J.H., Liu B., Tian H.Y., Yang W.P. (2020). The feeding of dietary Codonopsis pilosula polysaccharide enhances the immune responses, the expression of immune-related genes and the growth performance of red swamp crayfish (*Procambarus clarkii*). Fish Shellfish Immunol..

[5] He X., Fang J., Ruan Y., Wang X., Sun Y., Wu N., Zhao Z., Chang Y., Ning N., Guo H. (2018). Structures, bioactivities and future prospective of polysaccharides from Morus alba (white mulberry): A review. Food Chem..

[6] Jian F.X. (2009). The Regulation of Mouse Immune Functions by olysaccharides from Mulberry Bark. Sci. Seric..

[7] Guo C., Li R., Zheng N., Xu L., Liang T., He Q. (2013). Anti-diabetic effect of ramulus mori polysaccharides, isolated from Morus alba L., on STZ-diabetic mice through blocking inflammatory response and attenuating oxidative stress. Int. Immunopharmacol..

[8] Liu C.J., Lin J.Y. (2012). Anti-inflammatory and anti-apoptotic effects of strawberry and mulberry fruit polysaccharides on lipopolysaccharide-stimulated macrophages through modulating pro-/anti-inflammatory cytokines secretion and Bcl-2/Bak protein ratio. Food Chem. Toxicol..

[9] Zhang Y., Ren C., Lu G., Mu Z., Cui W., Gao H., Wang Y. (2014). Anti-diabetic effect of mulberry leaf polysaccharide by inhibiting pancreatic islet cell apoptosis and ameliorating insulin secretory capacity in diabetic rats. Int. Immunopharmacol..

[10] Banerjee P., Mukherjee S., Bera K., Ghosh K., Ali I., Khawas S., Ray S. (2019). Polysaccharides from *Thymus vulgaris* leaf: Structural features, antioxidant activity and interaction with bovine serum albumin. Int. J. Biol. Macromol..

[11] Tahmouzi S., Nejat M.R.S. (2020). New infertility therapy effects of polysaccharides from *Althaea officinalis* leaf with emphasis on characterization, antioxidant and anti-pathogenic activity. Int. J. Biol. Macromol..

[12] Chen R., Zhou X., Deng Q., Yang M., Li S., Zhang Q., Chen H. (2023). Extraction, structural characterization and biological activities of polysaccharides from mulberry leaves: A review. Int. J. Biol. Macromol..

[13] Chen X., Cai B., Wang J., Sheng Z., Yang H., Wang D., Chen J., Ning Q. (2021). Mulberry leaf-derived polysaccharide modulates the immune response and gut microbiota composition in immunosuppressed mice. J. Funct. Foods.

[14] Liu C., Ma Y., Zhang X. (2017). Effects of mulberry leaf polysaccharide on oxidative stress in pancreatic β-cells of type 2 diabetic rats. Eur. Rev. Med. Pharmacol. Sci..

[15] Ren C., Zhang Y., Cui W., Lu G., Wang Y., Gao H., Huang L., Mu Z. (2015). A polysaccharide extract of mulberry leaf ameliorates hepatic glucose metabolism and insulin signaling in rats with type 2 diabetes induced by high fat-diet and streptozotocin. Int. J. Biol. Macromol..

[16] Zhang Z., Li L., Li Z.K., Wu F., Hang B.Y., Cai B.Y., Du Y.G. (2018). Effect and mechanism of mulberry leaf polysaccharide on type 1 diabetic nephropathy in rats. Zhonghua Yi Xue Za Zhi.

[17] Voorhees R. (2020). Membrane Protein Biosynthesis at the Endoplasmic Reticulum. Microsc. Microanal..

[18] Zhang H., Hu W., Zhong Y., Guo Z. (2021). Meta-analysis of the effects of smooth endoplasmic reticulum aggregation on birth outcome. BMC Pregnancy Childbirth.

[19] Raote I., Malhotra V. (2021). Tunnels for protein export from the endoplasmic reticulum. Annu. Rev. Biochem..

[20] Wang Y., Hu H., Li H., Ma H., Xu F., Qu B. (2014). Effects of lead exposure on placental cellular apoptosis and endoplasmic reticulum stress in rats. Chin. Med. J..

[21] Wu K., Luo Z., Hogstrand C., Chen G.H., Wei C.C., Li D.D. (2018). Zn stimulates the phospholipids biosynthesis via the pathways of oxidative and endoplasmic reticulum stress in the intestine of freshwater teleost yellow catfish. Environ. Sci. Technol..

[22] Bravo R., Parra V., Gatica D., Rodríguez A.E., Torrealba N., Paredes F., Wang Z.V., Zorzano A., Hill J.A., Jaimovich E. (2013). Endoplasmic reticulum and the unfolded protein response: Dynamics and metabolic integration. Int. Rev. Cell Mol. Biol. Rep..

[23] Hu R., Wang S., Feng L., Jiang W., Wu P., Liu Y., Jin X., Kuang S., Tang L., Zhang L. (2023). Alleviation of hypoxia stress induced oxidative damage, endoplasmic reticulum stress (ERS) and autophagy in grass carp (*Ctenopharyngodon idellu*) by TTO (*Melaleuca alternifolia* essential oil). Aquaculture.

[24] Ou H., Liang J., Liu J. (2022). Effects of acute ammonia exposure on oxidative stress, endoplasmic reticulum stress and apoptosis in the kuruma shrimp (*Marsupenaeus japonicus*). Aquac. Rep..

[25] Fu C., Qiu Z., Huang Y., Lin Q., Jin L., Tu H., Ye J., Zheng C., Zhong W., Ma D. (2022). Achyranthes bidentata polysaccharides alleviate endoplasmic reticulum stress in osteoarthritis via lncRNA NEAT1/miR-377-3p pathway. Biomed. Pharmacother..

[26] Sun S., Yang S., An N., Wang G., Xu Q., Liu J., Mao Y. (2019). Astragalus polysaccharides inhibits cardiomyocyte apoptosis during diabetic cardiomyopathy via the endoplasmic reticulum stress pathway. J. Ethnopharmacol..

[27] Yang F., Wei Y., Liao B., Wei G., Qin H., Pang X., Wang J. (2020). Effects of Lycium barbarum Polysaccharide on Endoplasmic Reticulum Stress and Oxidative Stress in Obese Mice. Front. Pharmacol..

[28] Shelley L.K., Ross P.S., Kennedy C.J. (2012). The effects of an in vitro exposure to 17β-estradiol and nonylphenol on rainbow trout (*Oncorhynchus mykiss*) peripheral blood leukocytes. Comp. Biochem. Physiol. C Toxicol. Pharmacol..

[29] Liu B., Yang Z., Bo L., Zhao Z., Sun C. (2019). Cytotoxic effects, inflammatory response and apoptosis induction of cyclophosphamide in the peripheral blood leukocyte of blunt snout bream (*Megalobrama amblycephala*). Fish Shellfish Immunol..

[30] Huang X., Miao L.H., Lin Y., Pan W.J., Ren M.C., Ge X.P., Liu B., Zhou Q.L. (2018). High glucose affected respiratory burst activity of peripheral leukocyte via G6PD and NOX inhibition in *Megalobrama amblycephala*. Fish Shellfish Immunol..

[31] Betoulle S., Duchiron C., Deschaux P. (2000). Lindane differently modulates intracellular calcium levels in two populations of rainbow trout (*Oncorhynchus mykiss*) immune cells: Head kidney phagocytes and peripheral blood leucocytes. Toxicology.

[32] Hao H., Cao L., Jiang C., Che Y., Zhang S., Takahashi S. (2017). Farnesoid X Receptor Regulation of the NLRP3 Inflammasome Underlies Cholestasis-Associated Sepsis. Cell Metab..

[33] Du L., Lin L., Li Q., Liu K., Huang Y., Wang X. (2019). IGF-2 Preprograms Maturing Macrophages to Acquire Oxidative Phosphorylation-Dependent Anti-inflammatory Properties. Cell Metab..

[34] Chen S., Sun L., Hao M., Liu X. (2022). Circ-SWT1 Ameliorates H_2_O_2_-Induced Apoptosis, Oxidative Stress and Endoplasmic Reticulum Stress in Cardiomyocytes via miR-192-5p/SOD2 Axis. Cardiovasc. Toxicol..

[35] Jiang X., Liao X.H., Huang L.L., Sun H., Liu Q., Zhang L. (2019). Overexpression of augmenter of liver regeneration (ALR) mitigates the effect of H2O2-induced endoplasmic reticulum stress in renal tubule epithelial cells. Apoptosis.

[36] Jia R., Du J., Cao L., Feng W., He Q., Xu P. (2020). Chronic exposure of hydrogen peroxide alters redox state, apoptosis and endoplasmic reticulum stress in common carp (*Cyprinus carpio*). Aquat. Toxicol..

[37] Pobre K.F.R., Poet G.J., Hendershot L.M. (2019). The endoplasmic reticulum (ER) chaperone BiP is a master regulator of ER functions: Getting by with a little help from ERdj friends. J. Biol. Chem..

[38] Rao J., Yue S., Fu Y., Zhu J., Wang X., Busuttil R.W. (2014). ATF6 Mediates a Pro-Inflammatory Synergy Between ER Stress and TLR Activation in the Pathogenesis of Liver Ischemia-Reperfusion Injury. Am. J. Transplant..

[39] Zhou X., Lu B., Fu D., Gui M., Yao L., Li J. (2020). Huoxue Qianyang decoction ameliorates cardiac remodeling in obese spontaneously hypertensive rats in association with ATF6-CHOP endoplasmic reticulum stress signaling pathway regulation. Biomed. Pharmacother..

[40] Wu L., Li H., Xu W., Dong B., Geng H., Jin J., Han D., Liu H., Zhu X., Yang Y. (2022). Emodin alleviates acute hypoxia-induced apoptosis in gibel carp (*Carassius gibelio*) by upregulating autophagy through modulation of the AMPK/mTOR pathway. Aquaculture.

[41] Huang C., Yao R., Zhu Z., Pang D., Cao X., Feng B., Paulsen B.S., Li L., Yin Z., Chen X. (2019). A pectic polysaccharide from water decoction of Xinjiang Lycium barbarum fruit protects against intestinal endoplasmic reticulum stress. Int. J. Biol. Macromol..

[42] Gómez-Crisóstomo N.P., Rodríguez Martínez E., Selva R.A. (2014). Oxidative stress activates the transcription factors FoxO 1a and FoxO 3a in the hippocampus of rats exposed to low doses of ozone. Oxid. Med. Cell Longev..

[43] Shen M., Lin F., Zhang J., Tang Y., Chen W.K., Liu H. (2012). Involvement of the up-regulated FoxO1 expression in follicular granulosa cell apoptosis induced by oxidative stress. J. Biol. Chem..

[44] Ding H.R., Tang Z.T., Tang N., Zhu Z.Y., Liu H.Y., Pan C.Y., Hu A.Y., Lin Y.Z., Gou P., Yuan X.W. (2020). Protective properties of FOXO1 inhibition in a murine model of non-alcoholic fatty liver disease are associated with attenuation of ER stress and necroptosis. Front. Physiol..

[45] Safra M., Fickentscher R., Levi-Ferber M., Danino Y.M., Haviv-Chesner A., Hansen M., Juven-Gershon T., Weiss M., Henis-Korenblit S. (2014). The FOXO transcription factor DAF-16 bypasses ire-1 requirement to promote endoplasmic reticulum homeostasis. Cell Metab..

[46] Farag M.R., Alagawany M., Khalil S.R., Moustafa A.A., Mahmoud H.K., Abdel-Latif H.M. (2021). Astragalus membranaceus polysaccharides modulate growth, hemato-biochemical indices, hepatic antioxidants, and expression of HSP70 and apoptosis-related genes in *Oreochromis niloticus* exposed to sub-lethal thallium toxicity. Fish Shellfish Immunol..

[47] Liu H., Fang Y., Zou C. (2021). Pomelo polysaccharide extract inhibits oxidative stress, inflammation, and mitochondrial apoptosis of *Epinephelus coioides*. Aquaculture.

[48] Zuo Z., Wang S., Wang Q., Wang D., Wu Q., Xie S., Zou J. (2022). Effects of partial replacement of dietary flour meal with seaweed polysaccharides on the resistance to ammonia stress in the intestine of hybrid snakehead (*Channa maculatus* ♀ *× Channa argus* ♂). Fish Shellfish Immunol..

[49] Mohammadi G., Karimi A.A., Hafezieh M., Dawood M.A., Abo-Al-Ela H.G. (2022). Pistachio hull polysaccharide protects Nile tilapia against LPS-induced excessive inflammatory responses and oxidative stress, possibly via TLR2 and Nrf2 signaling pathways. Fish Shellfish Immunol..

[50] Fan H., Sun M., Li J., Zhang S., Tu G., Liu K., Xia Q., Jiang Y., Liu B. (2023). Structure characterization and immunomodulatory activity of a polysaccharide from Saposhnikoviae Radix. Int. J. Biol. Macromol..

[51] Xiao Z., Deng Q., Zhou W., Zhang Y. (2022). Immune activities of polysaccharides isolated from *Lycium barbarum* L. What do we know so far?. Pharm. Ther..

[52] Cao W., Wu J., Zhao X., Li Z., Yu J., Shao T., Han J. (2024). Structural elucidation of an active polysaccharide from *Radix Puerariae lobatae* and its protection against acute alcoholic liver disease. Carbohydr. Polym..

[53] Qin X., Nong K., Liu Z., Fang X., Zhang B., Chen W., Wang Z., Wu Y., Shi H., Wang X. (2024). Regulation of the intestinal flora using polysaccharides from *Callicarpa nudiflora* Hook to alleviate ulcerative colitis and the molecular mechanisms involved. Int. J. Biol. Macromol..

[54] Wang Z., Liu Y., Sun Y., Mou Q., Wang B., Zhang Y., Huang L. (2014). Structural characterization of LbGp1 from the fruits of *Lycium barbarum* L. Food Chem..

[55] Gong G., Dang T., Deng Y., Han J., Zou Z., Jing S., Zhang Y., Liu Q., Huang L., Wang Z. (2018). Physicochemical properties and biological activities of polysaccharides from Lycium barbarum prepared by fractional precipitation. Int. J. Biol. Macromol..

[56] Sun S., Li K., Xiao L., Lei Z., Zhang Z. (2019). Characterization of polysaccharide from Helicteres angustifolia L. and its immunomodulatory activities on macrophages RAW264. 7. Biomed. Pharmacother..

[57] Csordás G.R., Renken C., Várnai P., Walter L., Weaver D., Buttle K.F., Balla T., Mannella C.A., Hajnóczky G.R. (2006). Structural and functional features and significance of the physical linkage between ER and mitochondria. J. Cell Biol..

[58] Peng J., Peng C., Wang L., Cao H., Xing C., Li G., Hu G., Yang F. (2022). Endoplasmic reticulum-mitochondria coupling attenuates vanadium-induced apoptosis via IP3R in duck renal tubular epithelial cells. J. Inorg. Chem..

[59] De Brito O.M., Scorrano L. (2008). Mitofusin 2 tethers endoplasmic reticulum to mitochondria. Nature..

[60] Erustes A.G., D’Eletto M., Guarache G.C., Ureshino R.P., Bincoletto C., Silva Pereira G.J., Piacentini M., Smaili S.S. (2021). Overexpression of α-synuclein inhibits mitochondrial Ca^2+^ trafficking between the endoplasmic reticulum and mitochondria through MAMs by altering the GRP75–IP3R interaction. J. Neurosci. Res..

[61] Wang W., Xie Q., Zhou X., Yao J., Zhu X., Huang P., Zhang L., Wei J., Xie H., Zhou L. (2015). Mitofusin-2 triggers mitochondria Ca^2+^ influx from the endoplasmic reticulum to induce apoptosis in hepatocellular carcinoma cells. Cancer Lett..

[62] Misrani A., Tabassum S., Yang L. (2021). Mitochondrial Dysfunction and Oxidative Stress in Alzheimer’s Disease. Front. Aging Neurosci..

[63] Sharma C., Kim S., Nam Y., Jung U.J., Kim S.R. (2021). Mitochondrial Dysfunction as a Driver of Cognitive Impairment in Alzheimer’s Disease. Int. J. Mol. Sci..

[64] Xiang Q.D., Yu Q., Wang H., Zhao M.M., Liu S.Y., Nie S.P., Xie M.Y. (2017). Immunomodulatory Activity of Ganoderma atrum Polysaccharide on Purified T Lymphocytes through Ca^2+^/CaN and Mitogen-Activated Protein Kinase Pathway Based on RNA Sequencing. J. Agric. Food Chem..

[65] Miao X.Y., Zhu X.X., Gu Z.Y., Fu B., Cui S.Y., Zu Y., Rong L.J., Hu F., Chen X.M., Gong Y.P. (2022). *Astragalus* polysaccharides reduce high-glucose-induced rat aortic endothelial cell senescence and inflammasome activation by modulating the mitochondrial Na^+^/Ca^2+^ exchanger. Cell Biochem. Biophys..

[66] Sun S.L., Guo L., Ren Y.C., Wang B., Li R.H., Qi Y.S., Yu H., Chang N.D., Li M.H., Peng H.S. (2014). Anti-apoptosis effect of polysaccharide isolated from the seeds of *Cuscuta chinensis* Lam on cardiomyocytes in aging rats. Mol. Biol. Rep..

[67] Dai H., Jia G., Liu X., Liu Z., Wang H. (2014). Astragalus polysaccharide inhibits isoprenaline-induced cardiac hypertrophy via suppressing Ca^2+^-mediated calcineurin/NFATc3 and CaMKII signaling cascades. Environ. Toxicol. Pharmacol. Ther..

[68] Hu S., Wang D., Zhang J., Du M., Cheng Y., Liu Y., Zhang N., Wang D., Wu Y. (2016). Mitochondria related pathway is essential for polysaccharides purified from *sparassis crispa* mediated neuro-protection against glutamate-induced toxicity in differentiated PC12 cells. Int. J. Mol. Sci..

[69] Russo A., Cardile V., Graziano A.C., Avola R., Bruno M., Rigano D. (2018). Involvement of Bax and Bcl-2 in induction of apoptosis by essential oils of three Lebanese Salvia species in human prostate cancer cells. Int. J. Mol. Sci..

[70] Zhou H., Wang X., Zhang B. (2020). Depression of lncRNA NEAT1 antagonizes LPS-evoked acute injury and inflammatory response in alveolar epithelial cells via HMGB1-RAGE signaling. Mediat. Inflamm..

[71] Suski J.M., Lebiedzinska M., Bonora M., Pinton P., Duszynski J., Wieckowski M.R. (2018). Relation between mitochondrial membrane potential and ROS formation. Mitochondrial Bioenergetics: Methods Protocols.

[72] Duan Y., Gross R.A., Sheu S.S. (2007). Ca^2+^-dependent generation of mitochondrial reactive oxygen species serves as a signal for poly (ADP-ribose) polymerase-1 activation during glutamate excitotoxicity. J. Physiol..

[73] Halliwell B., Gutteridge J.M. (2015). Free Radicals in Biology and Medicine.

[74] Cui Y.T., Liu B., Xie J., Xu P., Habte-Tsion H., Zhang Y.Y. (2014). The effect of emodin on cytotoxicity, apoptosis and antioxidant capacity in the hepatic cells of grass carp (*Ctenopharyngodon idellus*). Fish Shellfish Immunol..

[75] Yi J., Li X., Wang S., Wu T., Liu P. (2022). Steam explosion pretreatment of *Achyranthis bidentatae* radix: Modified polysaccharide and its antioxidant activities. Food Chem..

[76] Zhu X., Liu X., Xia C., Li M., Niu X., Wang G., Zhang D. (2021). Effects of dietary *Astragalus Propinquus Schischkin* polysaccharides on growth performance, immunological parameters, antioxidants responses and inflammation-related gene expression in *Channa argus*. Comparative biochemistry and physiology. Toxicol. Pharmacol..

[77] Shi F.L., Zhi J.Y., Min X.L., Feng Z., Fan B.Z., Li J.L., Yan A.L., Qing Q.L., Jiang T.L., Jun L. (2021). Astragalus polysaccharides mediate the immune response and intestinal microbiota in grass carp (*Ctenopharyngodon idellus*). Aquaculture.

